# High nitrogen use efficiency in wheat is explained by a longer fast-increase period and adequate pre-anthesis nitrogen accumulation

**DOI:** 10.3389/fpls.2025.1727679

**Published:** 2026-01-23

**Authors:** Minglong Yu, Churong Liu, Hongrun Liu, Yushi Zhang, Zhaohu Li, Mingcai Zhang

**Affiliations:** 1 Engineering Research Center of Plant Growth Regulator, Ministry of Education, College of Agronomy and Biotechnology, China Agricultural University, Beijing, China; 2 Guangdong Academy of Sciences, Institute of Nanfan & Seed Industry, Guangzhou, China; 3 Key Laboratory of Agricultural Green and Low Carbon, Ministry of Agriculture and Rural Affairs, Beijing, China

**Keywords:** fast-increase period, grain filling, nitrogen accumulation, nitrogen responsiveness, wheat

## Abstract

**Introduction:**

Nitrogen (N)-efficient wheat cultivars achieve higher grain yields with equivalent N fertilizer inputs, and the grain filling character largely determines grain weight (GW) in cereal crops. However, the relationship of grain filling traits and N responsiveness (N_r_) in wheat has not been fully evaluated.

**Methods:**

A two-year field experiment evaluated five wheat cultivars across varying N levels (0, 75, 150, and 225 kg N ha^−1^) to assess how grain filling traits and N-related characteristics influence N_r_.

**Results:**

The results showed that N-responsiveness wheat cultivars exhibited higher grain yields and critical N supply, alongside lower chlorophyll degradation rates (CDR). The direct path coefficient of GW on yield was 0.478, which explained 85.2% of the yield variation and was negatively correlated with other yield components. Across the combinations of cultivar and N supply, the variation in GW was primarily driven by the duration of fast-increase period (T_fast_), rather than by the duration of slow-increase period (*T*_slow_) and slight-increase period (T_slight_). Furthermore, the sensitivity of *T*_fast_ to N supply explained the N_r_ of grain yield in wheat. Structural equation modeling showed that adequate pre-anthesis N accumulation was the dominant factor driving the extension of *T*_fast_ in high N-responsiveness wheat cultivates, secondary to lower CDR, which ultimately resulted in the highest GW. In addition, prolonging *T*_fast_ induced enhanced post-anthesis N translocation in wheat, which contributed to higher N use efficiency (NUE).

**Discussion:**

Prolonging the *T*_fast_ enhances N responsiveness in wheat grain yield, providing a novel framework for evaluating NUE. This finding also highlights the critical role of elevated N accumulation at anthesis under N fertilization.

## 1 Introduction

Wheat (*Triticum aestivum* L.) is a cornerstone of global food security, supplying over 20% of humanity’s dietary calories and protein. However, its production comes at a significant environmental cost, as it is the world’s second-largest consumer of nitrogen (N) fertilizer, accounting for 18.3% of global N use (IFA, 2022). Genetic improvement in wheat increases yield, especially at high N supply, i.e., wheat breeding improves N responsiveness (N_r_) (Lian et al., 2023; Peng et al., 2022; Tian et al., 2011). This indicated that breeding is advancing toward N-efficient cultivars, as N-efficient cultivars tend to be higher N_r_ (Aga et al., 2022; Ding et al., 2023). N-efficient wheat cultivars not only achieve higher economic yields based on higher N use efficiency (NUE) but also alleviate environmental problems caused by excess N supply compared to other cultivars (Ying et al., 2019). Therefore, defining plant traits associated with N efficiency will further accelerate the pace of breeding.

Grain number is determined by resource capture during pre-anthesis and anthesis (source limitation), whereas grain weight (GW) depends more critically on source-sink balance during grain filling (Akin et al., 2017; Cao et al., 2019; Slafer et al., 2022; Zhou et al., 2018). The final GW is predominantly governed by the dynamics of grain filling–a process that can be quantitatively dissected into distinct phases: the slow-increase period (*T*_slow_), fast-increase period (*T*_fast_), and slight-increase period (*T*_slight_) (Bancal, 2009; Li et al., 2020; Zhou et al., 2018). N-efficient modern cultivars demonstrate accelerated grain filling rate (*GFR*) through shortened *T*_slow_, while extended *T*_fast_ and *T*_slight_ collectively generate longer grain filling duration (*GFD*) (Zhang et al., 2024). Previous studies have pointed out that *GFR* and *GFD* are thought to be regulated by genetic factors, while the latter is simultaneously sensitive to environmental factors (e.g., N application, planting density, and temperature) (Li et al., 2020; Ma et al., 2022). The N supply level critically influences wheat yield. While N deficiency induces premature senescence (Barraclough et al., 2014; Lian et al., 2023), an optimal N application not only delays leaf senescence, but also prolongs *GFD* and promotes remobilization of assimilates to increase GW and yield (Du et al., 2024; Wang et al., 2024). Conversely, excessive N inhibits grain sink strength and the *GFR*, ultimately reducing yield potential (Zhao et al., 2022). Nevertheless, the potential linkage between N-induced variations in *GFD* (including *T*_slow_, *T*_fast_, and *T*_slight_) and wheat’s N_r_ (i.e., N efficient) remains poorly investigated. Previous studies of grain filling traits has frequently been conducted under single or optimal N conditions, neglecting how variable N supply differentially modulates specific grain filling parameters. Consequently, it remains unresolved which specific grain filling phase (e.g., *T*_fast_) is most sensitive to N supply, and whether genotypic variation in the plasticity of this phase forms the physiological basis for differences in N_r_. Therefore, elucidating this link is critical for developing targeted breeding strategies aimed at N-efficient cultivars. In addition, continuous monitoring of leaf SPAD values during grain filling could accurately reflect leaf N sinks and chlorophyll degradation rate (CDR), and has been widely used in modern smart agriculture to invert the growth status of plants at different reproductive periods for effective nutrient management (Mehrabi and Sepaskhah, 2022; Shen et al., 2022; Yang et al., 2021). Barraclough et al. (2014) emphasized that remobilization of stored N in preference to photosynthetic N from vegetative tissues helps delay leaf senescence while meeting the demand for high N grains. It seems reasonable to assume that high-yielding and N-efficient wheat cultivars are usually accompanied by larger N source during grain filling, which allows N accumulated at anthesis to be matched with a stronger rate and duration as well as lower CDR during grain filling. In other words, N is translocated more efficiently while maintaining longer transit times, which contributes to increased NUE (Tian et al., 2011, 2016). While some studies suggest that enhanced N accumulation at either individual or population levels drives improved filling capacity in N-efficient wheat cultivars under increased N supply (Bancal, 2009), quantitative evidence linking specific grain filling traits to N accumulation remains limited.

The NUE is governed by both uptake and translocation processes, with these mechanisms showing strong dependence on N availability (Barraclough et al., 2010; Cassman et al., 2002; Li et al., 2020). Modern wheat cultivars exhibit an enhanced response to high N supply, leading to concurrent improvements in both N uptake efficiency (NUpE) and N utilization efficiency (NUtE), thereby contributing to overall NUE gains through breeding (Hu et al., 2023; Ren et al., 2023; Sadras et al., 2016; Tian et al., 2016). Previous investigations have revealed that the majority (70-95%) of grain N allocation stems from pre-anthesis accumulation, with post-anthesis uptake supplying the remainder (Barraclough et al., 2010). Therefore, N translocation during grain filling stage may play a decisive role in NUE. However, it remains little documented whether genetic differences in wheat grain filling affect N transport during this stage, and whether this is related to N_r_. This two-year field study evaluated five winter wheat cultivars with varying N_r_ across four N application rates. This study aimed to: (i) clarify the essential role of N-induced grain filling traits on GW establishment, (ii) analyze the critical grain filling factors driving high N use efficiency in wheat, and (iii) elucidate the N-related physiological drivers (e.g., pre-anthesis N accumulation, chlorophyll degradation) that modulate this critical grain filling phase. We hypothesized that the sensitivity of the *T*_fast_ to N supply is the primary determinant of a cultivar’s N_r_, and that this sensitivity is intrinsically linked to superior pre-anthesis N accumulation. This study aims to bridge the gap between N management and grain filling physiology, providing a novel mechanistic perspective for improving N use efficiency in wheat.

## 2 Materials and methods

### 2.1 Experimental location

**Figure 1:**
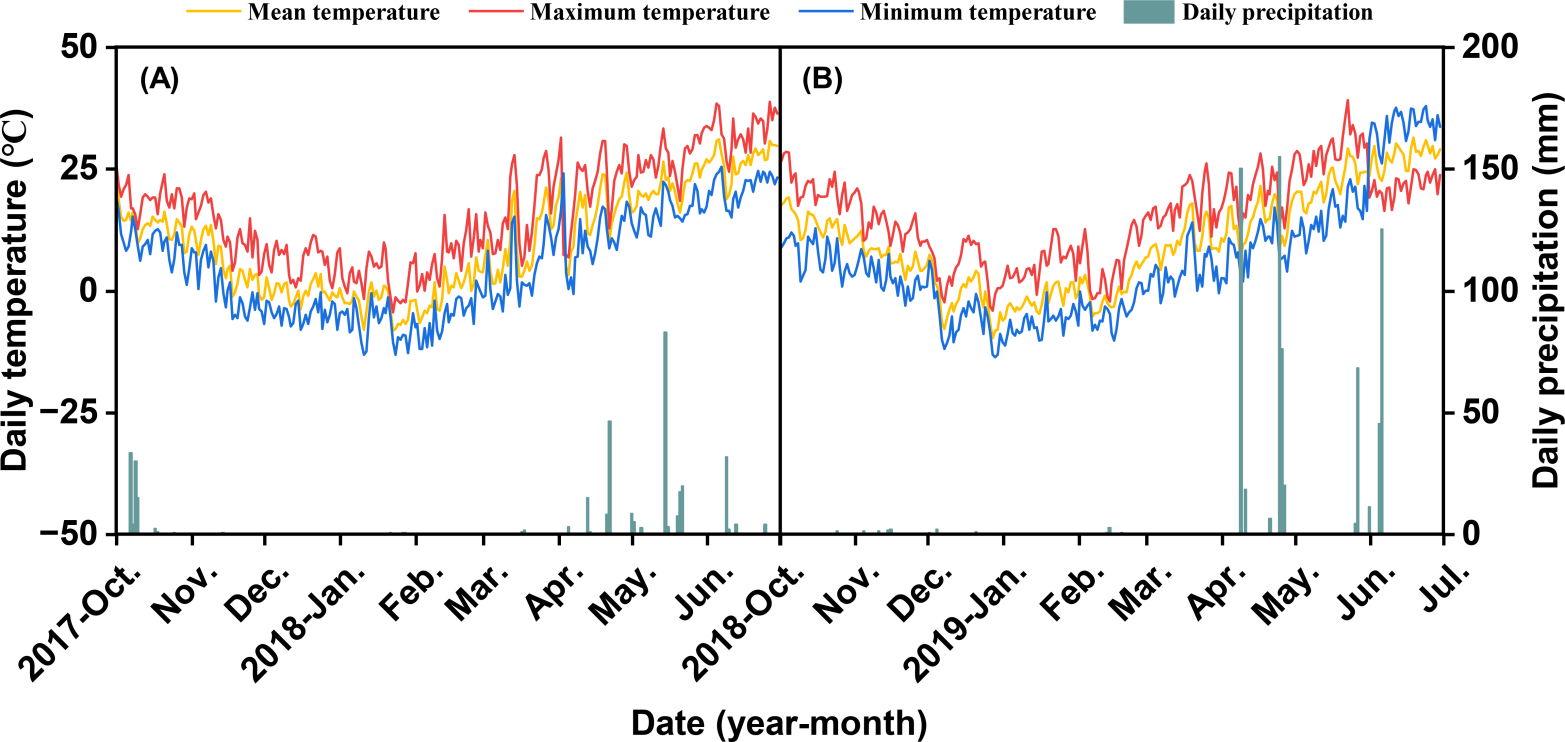
Daily maximum temperature, minimum temperature, mean temperature, and precipitation during the winter wheat growing season in Wuqiao in 2017-2018 **(A)** and 2018-2019 **(B)**.

The field trials were conducted from 2017-2019 (two growing seasons) in the Wuqiao Experimental Station of China Agricultural University (Wuqiao County, Hebei Province, 37° 63′ N, 116° 28′ E), located the North China Plain. This region exhibits a temperate semi-arid monsoon climate with mean temperatures of 10.0 °C and 10.2 °C and precipitation of 346.4 mm and 689.4 mm for the growing seasons of 2017–2018 and 2018-2019, respectively (**Figure 1**). The experimental site is a typical winter wheat-summer maize cropping system area. The soil is classified as fluvo-aquic soil with sandy clay loam in the top soil. The tillage layer of 0–20 cm contains 0.74 g kg^−1^ of total N, 18.8 mg kg^−1^ of Olsen-P, 127.3 mg kg^−1^ of available-K, 12.2 g kg^−1^ of organic matter, and pH 8.2.

### 2.2 Experimental design

A split-plot design with three replicates was employed, where N supply served as the main plots and cultivars constituted the sub-plots. Urea (46% N) applied as the N fertilizer at four rates: 0 (N0), 75 (N75), 150 (N150), and 225 kg N ha^−1^ (N225), with a 1:1 ratio at sowing and jointing. Five wheat cultivars, BiMa1 (BM1), JiNan2 (JN2), TaiShan1 (TS1), JiMai26 (JM26), and JiMai22 (JM22), released in 1950s, 1960s, 1970s, 1980s, and 2000s respectively, were used as experimental materials. These cultivars exhibit varying N responsiveness in yield and were widely cultivated in their respective eras (Chen et al., 2019; Yang et al., 2013). The experimental plots measured 10.8 m² (6.0 × 1.8 m), containing 12 rows each 6 m length with 0.15 m row spacing. A 1 m buffer zone separated N treatment plots to reduce cross-plot interference (Hu et al., 2023; Shen et al., 2025; Zheng et al., 2022). Using mechanical sowing, seeds were planted at a rate of 225 kg ha^−1^ on October 12, 2017, and October 11, 2018, with corresponding harvest dates of June 12, 2018, and June 12, 2019, respectively. Prior to sowing, each plot received basal fertilizer applications of 90 kg ha^−1^ P_2_O_5_ (calcium superphosphate, 12% P_2_O_5_) and 90 kg ha^−1^ K_2_O (potassium sulfate, 50% K_2_O), which were uniformly broadcast across all treatment areas. During the 2018 growing season, three irrigation events were conducted–prior to winter dormancy, during jointing, and at anthesis–with each application delivering approximately 60 mm of water. Differently, due to high precipitation (> 60 mm) in April 2019, only two irrigations of 60 mm each were performed - before winter dormancy and at anthesis. Chemical controls effectively managed pests and diseases throughout the growing season, with no significant occurrences of weeds, pests, or disease observed in any subplot.

### 2.3 Plant sampling and determination methods

#### 2.3.1 Grain yield, grain weight and nitrogen responsiveness

At physiological maturity, wheat plants from a 1 m^2^ area were manually harvested in each plot, and the samples were subsequently weighed separately after threshing and natural drying to determine grain yield (GY) at 13% moisture content. Meanwhile, twenty spikes were randomly selected from each plot to count the grains per spike (GPS) and calculate the grain weight (GW, 1000 grain) at physiological maturity. In addition, the spikes per unit area (SPUA) were counted at milk stage. The relationship between N supply and GY was accurately fitted by a “linear + plateau” model. Briefly, the slope of the linear phase was used to characterize the N responsiveness (N_r_) of cultivars, and the minimum N application rate needed to achieve maximum grain yield (GY_max_) was defined as the critical N supply (N_cs_).

#### 2.3.2 Grain filling traits

For each plot, 30 tagged wheat ears were sampled every six days from anthesis until 36 days post-anthesis. The grains were extracted, heat-fixed at 105 °C for 30 minutes, and subsequently dried at 75 °C to achieve a stable weight. The grain filling dynamics were calculated using the Richards (1959) growth equation:

(1) $$W\;=\;\frac{A}{{\left(1\;+\;Be^{-kt}\right)}^{\frac{1}{N}}}$$

where *W*, grain weight (mg); *A*, final grain weight (mg); *t*, days post-anthesis (d); *B*, *k*, and *N*, regression-derived coefficients.

The first and second derivatives of Equation 1 were calculated following the method of Ma et al. (2022), from which the following parameters were obtained through Equations 2–9:

Maximum grain filling rate (*GFR*_max_):

(2) $$GFR_{\max}\;=\;KA{\left(N\;+\;1\right)}^{\frac{-N\;-\;1}{N}}$$

Mean grain filling rate (*GFR*_mean_):

(3) $$GFR_{mean}\;=\;\frac{AK}{2(N\;+\;2)}$$

The end time of the slow-increase period (*T*_1_):

(4) $$T_{1}\;=\;\frac{-\ln\frac{N^{2}\;+\;3N\;+\;N\sqrt{N^{2}\;+\;6N\;+\;5}}{2B}}{K}$$

The end time of the fast-increase period (*T*_2_):

(5) $$T_{2}\;=\;\frac{-\ln\frac{N^{2}\;+\;3N\;-\;N\sqrt{N^{2}\;+\;6N\;+\;5}}{2B}}{K}$$

The end time of slight-increase period (*T*_3_):

(6) $$T_{3}\;=\;\frac{-\ln\left[\frac{{\left(\frac{100}{99}\right)}^{N}\;-\;1}{B}\right]}{K}$$

Duration of slow-increase period (*T*_slow_):

(7) $$T_{slow}=T_{1}$$

Duration of fast-increase period (*T*_fast_):

(8) $$T_{fast}=T_{2}-T_{1}$$

Duration of slight-increase period (*T*_slight_):

(9) $$T_{\text{slight}}=T_{3}–T_{2}$$

#### 2.3.3 SPAD and chlorophyll degradation rate

Flag leaf SPAD values was measured at 7, 14, 21, and 28 days after anthesis using a SPAD-502 meter (Minolta Camera Co., Ltd., Japan) to monitor senescence progression.

SPAD was fitted using Equation 10, Equation 11 is the first-order derivative of Equation 10, and the absolute value of the slope of Equation 11, |2a|, represents the mean linear decline rate calculated from the slope of a linear regression of SPAD against time for the post-anthesis period, serving as an indicator of the chlorophyll degradation rate (CDR).

(10) $$\text{y}=\text{a}{\text{x}}^{2}+\text{bx}+\text{c}$$

(11) y=2ax+b

#### 2.3.4 NUE, nitrogen nutrition index, nitrogen accumulation and translocation

At both anthesis and maturity stages, 20 wheat plants per plot were harvested and dissected into stems, leaves, chaffs, and grains (only at maturity). All organ samples were first heat-treated at 105 °C for 30 mins, then oven-dried at 80 °C until reaching constant mass, and finally weighed to determine the total shoot biomass. After grinding, N content was analyzed using a Vario Micro Cube elemental analyzer (Elementar, Germany) via the Dumas combustion method. Total N accumulation amount (N_TAA_) represents the cumulative N content across all plant organs, calculated according to Equation 12:

(12) $${\text{N}}_{\text{TAA}}\left(\text{g }{\text{m}}^{-2}\right)={\text{N}}_{\text{stem}}+{\text{N}}_{\text{chaff}}+{\text{N}}_{\text{leaf}}+{\text{N}}_{\text{grain}}$$

The nitrogen nutrition index (NNI), which is the ratio of the actual shoot N concentration (NC_act_) to the critical N concentration (NC_cri_), was calculated according to Equation 13. The critical concentration followed the function NC_cri_ = 4.59 × *W*_shoot_−0.41 (Blandino et al., 2022; Yang et al., 2023):

(13) $$\text{NNI}=\text{N}{\text{C}}_{\text{act}}/\text{N}{\text{C}}_{\text{cri}}$$

Where *W*_shoot_ is the total shoot biomass of wheat at anthesis stage, expressed in kg ha^−1^.

The following N-related parameters were calculated using Equations 14–19 as described by Peng et al. (2022):

(14) $$\text{N utilization efficiency}\left(\text{NUtE}\right)=\text{GY}/{\text{N}}_{\text{am}}$$

(15) $$\text{N uptake efficiency}\left(\text{NUpE}\right)={\text{N}}_{\text{am}}/{\text{N}}_{\text{e}}$$

(16) $$\text{N use efficiency}\left(\text{NUE}\right)=\text{GY}/{\text{N}}_{\text{e}}$$

(17) $$\text{N translocation amount}\left(\text{NTA}\right)\;={\text{N}}_{\text{aa}}-{\text{N}}_{\text{vtm}}$$

(18) $$\text{N translocation efficiency}\left(\text{NTE}\right)=\text{NTA}/{\text{N}}_{\text{aa}}\times 100$$

(19) $$\begin{array}{l} \text{Contribution rate of N translocation amount to grain }\\ \left(\text{CNTA}\right)=\text{NTA}/{\text{N}}_{\text{gm}}\times 100 \end{array}$$

Where GY, grain yield (t ha^−1^); N_am_, aboveground N accumulation amount at maturity (g m^−2^); N_e_, effective N supply [fertilizer N+ available N in 0–30 cm soil layer] (kg ha^-1^); N_aa_, aboveground N accumulation amount per unit area at anthesis (g m^−2^); N_vtm_, vegetative tissue N accumulation amount per unit area at maturity (g m^−2^); and N_gm_, grain N accumulation amount per unit area at maturity (g m^−2^).

### 2.4 Statistical analysis

The sensitivity of grain filling parameters, NTA, and N_TAA_ at anthesis to N supply was estimated using Equation 20 and expressed as slope k.

(20) y=kx+b

Analysis of variance was performed using SPSS 26.0 (IBM Corp., Chicago, IL, USA) to test the effects of N treatments, year, cultivars, and their interactions on yield components, grain filling characteristics, and N-related traits with least significant difference (Duncan’s) tests at 0.05, 0.01, or 0.001. All graphical representations were generated using Origin 2019 (Systat Software, San Jose, CA, USA). The contribution of grain filling traits to GW was investigated using random forest model (RFM, R package “randomForest”). Principle component analysis (PCA) and structural equation model (SEM) were conducted using Origin 2019 and Amos 24.0 (IBM Corp., Chicago, IL, USA) to gain insight into the relationships among yield components, grain filling traits, and N-related traits.

## 3 Result

### 3.1 The differences of N responsiveness in wheat cultivars subjected to N supply

**Figure 2:**
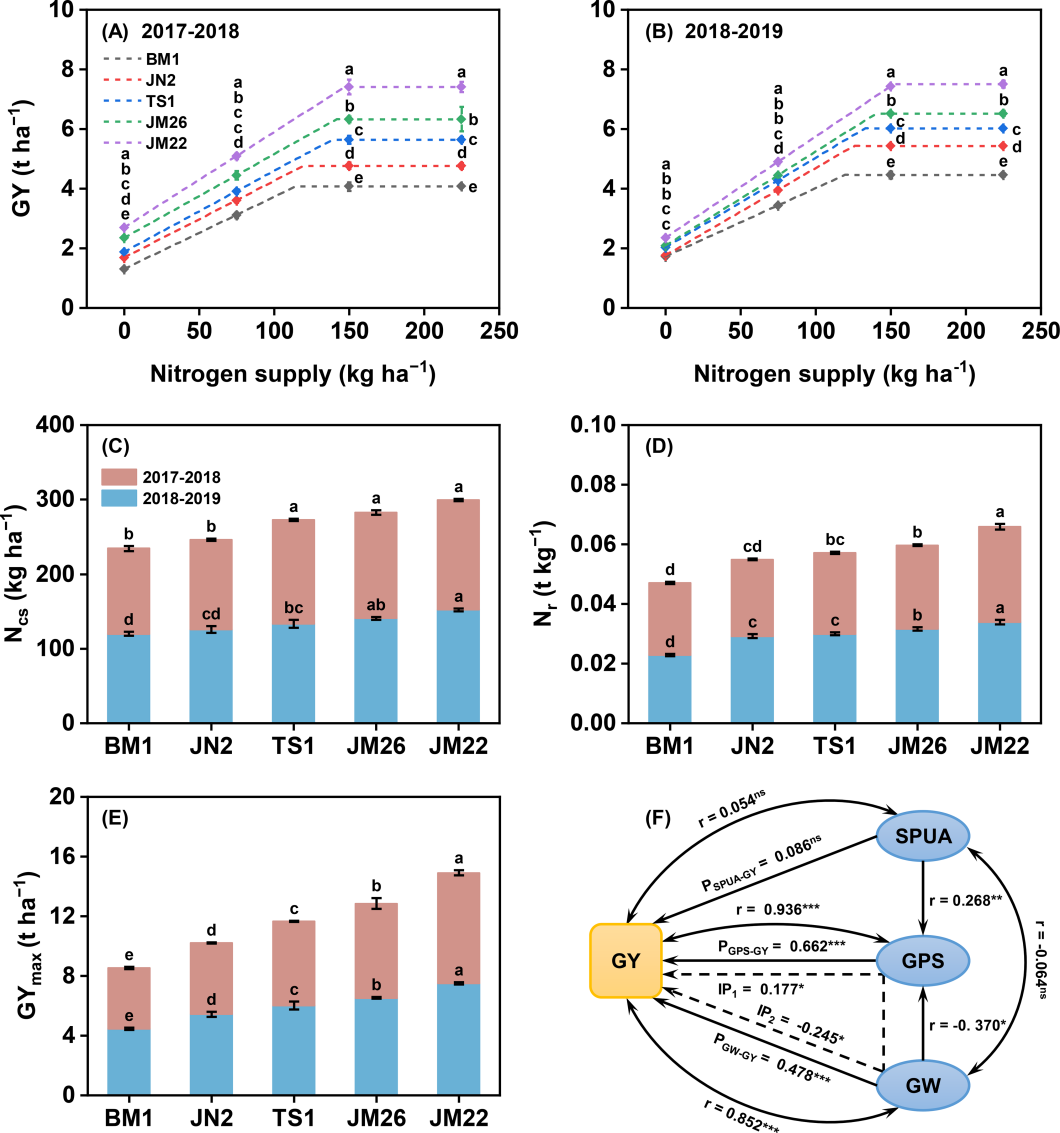
GY response of wheat cultivars to N supply in 2017-2018 **(A)** and 2018-2019 **(B)**. Differences of N_cs_**(C)**, N_r_**(D)**, and GY_max_**(E)** among wheat cultivars. Path coefficients of grain yield influenced by yield components under different N supply **(F)**. GY, grain yield; N_cs_, critical N supply; N_r_, N responsiveness; GY_max_, maximum grain yield; GW, grain weight; GPS, grains per spike; SPUA, spikes per unit area. In the path diagram: double-headed arrows (r) represent correlation coefficients; single-headed arrows indicate direct path coefficients; single-headed dashed arrows show indirect path coefficients; IP_1_, indirect path coefficient from GW to GY via GPS; and IP_2_, indirect path coefficient from GPS to GY via GW. Values represented mean ± SE, and different lowercase letters indicated significant differences between treatments at *P* < 0.05. *** indicated *P* < 0.001, ** indicated *P* < 0.01, * indicated *P* < 0.05, and ns indicated no significant difference.

Across the five cultivars, GY increased linearly at first and then remained constant when N supply was increased from 0 to 225 kg N ha^−1^ (**Figures 2A, B**). The GY, N_cs_, N_r_, and GY_max_ among the cultivars followed the order of JM22 > JM26 > TS1 > JN2 > BM1 (**Figures 2A−E**). In 2017-2018, the N_r_, N_cs_, and GY_max_ of JM22 increased 13.9-32.8%, 3.8-28.3%, and 17.1-82.1%, respectively, compared with the other cultivars. A similar pattern was observed in 2018-2019. Additionally, path analysis revealed strong positive correlations of GW (*P* < 0.001) and GPS (*P* < 0.001) with GY, irrespective of SPUA (**Figure 2F**). GW exhibited a strong direct effect on GY (path coefficient = 0.478, *P* < 0.001). GPS and GW also contributed significantly to GY through indirect pathways. Therefore, the variation in GY could be explained by GW and GPS, while this study mainly focused on the response of GW to yield formation. GW explained 90.4% of the variation in GY across cultivars and N supply. Meanwhile, the interaction effect between cultivar and N supply were significant on GY and GW (**Supplementary Table S1**).

### 3.2 Grain filling traits in response to N supply related to N responsiveness among wheat cultivars

**Figure 3:**
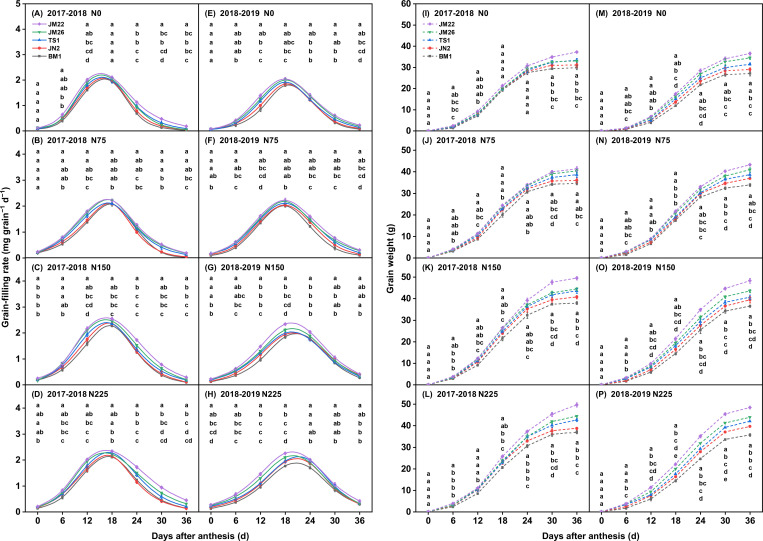
Effects of N supply on grain weight and grain filling rate among wheat cultivars. **(A–D)** represented the grain weight under 0, 75, 150, and 225 kg N ha^−1^ in 2017-2018, respectively. **(E–H)** represented the grain weight under 0, 75, 150, and 225 kg N ha^−1^ in 2018-2019, respectively. **(I–L)** represented the grain filling rate under 0, 75, 150, and 225 kg N ha^−1^ in 2017-2018, respectively. **(M–P)** represented the grain filling rate under 0, 75, 150, and 225 kg N ha^−1^ in 2018-2019, respectively. Values represented mean ± SE, and different lowercase letters indicated significant differences between treatments at *P* < 0.05.

N application increased GW across all wheat cultivars, and the order of GW was JM22 > JM26 > TS1 > JN2 > BM1 in both growing seasons under the same N supply level conditions (**Figures 3A−H**). At equivalent N application rates, JM22 exhibited the highest mean grian filling rate (*GFR*_mean_) and maximum grian filling rate (*GFR*_max_) among these cultivars. Compared with other cultivars, its *GFR*_mean_ and *GFR*_max_ were 0.7-14.8% and 0.5-11.4% higher in 2017-2018, and 3.4-26.0% and 3.0-21.5% higher in 2018-2019, respectively. Furthermore, JM22 displayed a shortened *T*_slow_ and an earlier onset along with extended *T*_fast_ and *T*_slight_ (**Figures 3I−P**, **Supplementary Table S2**). In contrast, BM1 showed a longer *T*_slow_ and shorter *T*_fast_ and *T*_slight_, resulting in the lowest *GFR*_mean_ and *GFR*_max_ among the cultivars.

**Figure 4:**
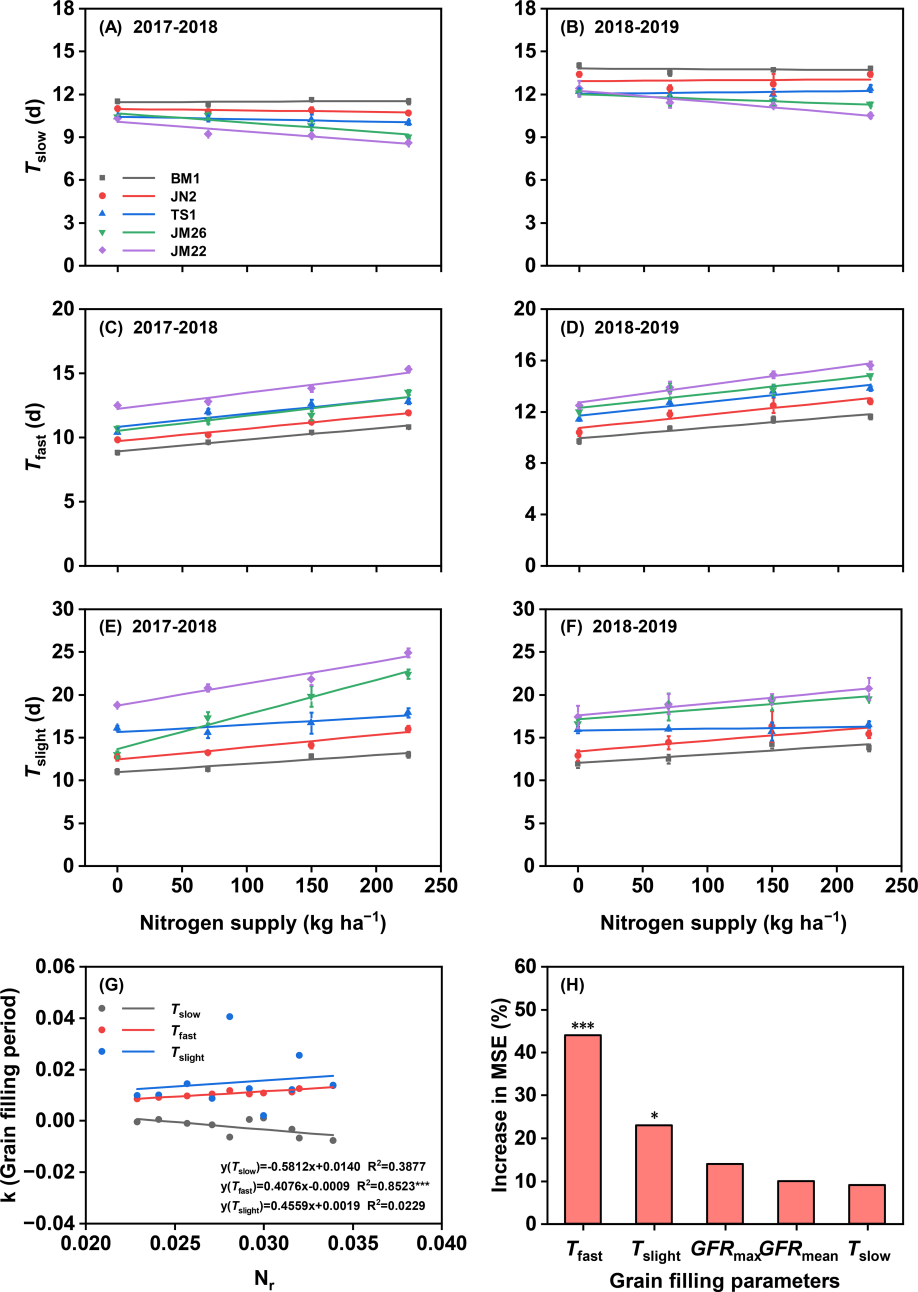
Response of grain filling parameters to N supply and its relationship with nitrogen responsiveness and grain weight. Duration of slow-increase period (*T*_slow_) in 2017-2018 **(A)** and 20218-2019 **(B)**, duration of fast-increase period (*T*_fast_) in 2017-2018 **(C)** and 2018-2019 **(D)**, duration of slight-increase period (*T*_slight_) in 2017-2018 **(E)** and 2018-2019 **(F)**. **(G)** Correlation between nitrogen responsiveness (N_r_) and sensitivity of grain filling period to N supply. **(H)** Random forest model (RFM) analysis of grain filling parameters revealed their relative contributions to GW development. Variable importance was assessed through percentage increase in mean squared error (%IncMSE), with larger values indicating stronger influence on GW prediction accuracy. k, sensitivity of grain filling period to N supply; *GFR*_max_, maximum grain filling rate; *GFR*_mean_, mean grain filling rate. Values represented mean ± SE. *** indicated *P* < 0.001, ** indicated *P* < 0.01, * indicated *P* < 0.05, and ns indicated no significant difference.

The N supply did not change *T*_slow_ in BM1, JN2, and TS1 in two growing seasons, but *T*_slow_ was reduced with increasing N supply in JM26 and JM22 (**Figures 4A, B**). Moreover, the *T*_slow_ of JM22 was the shortest at the same N rate among these cultivars while BM1 was the longest. The *T*_fast_ and *T*_slight_ were raised with increasing N supply among these cultivars, and the *T*_fast_ and *T*_slight_ of JM22 were consistently longer than other cultivars, while BM1 was the shortest at the same N rate among these cultivars (**Figures 4C−F**). Importantly, the N_r_ was positively correlated with the sensitivity of *T*_fast_ to N supply (*P <* 0.001), but independent of the sensitivity of *T*_slow_ and *T*_slight_ to N supply (**Figure 4G**, **Supplementary Table S3**). Similarly, the results of the RFM identified *T*_fast_ (*P* < 0.001) as the main driving factor affecting GW (**Figure 4H**).

### 3.3 N accumulation related to grain filling period under different N levels supply

**Figure 5:**
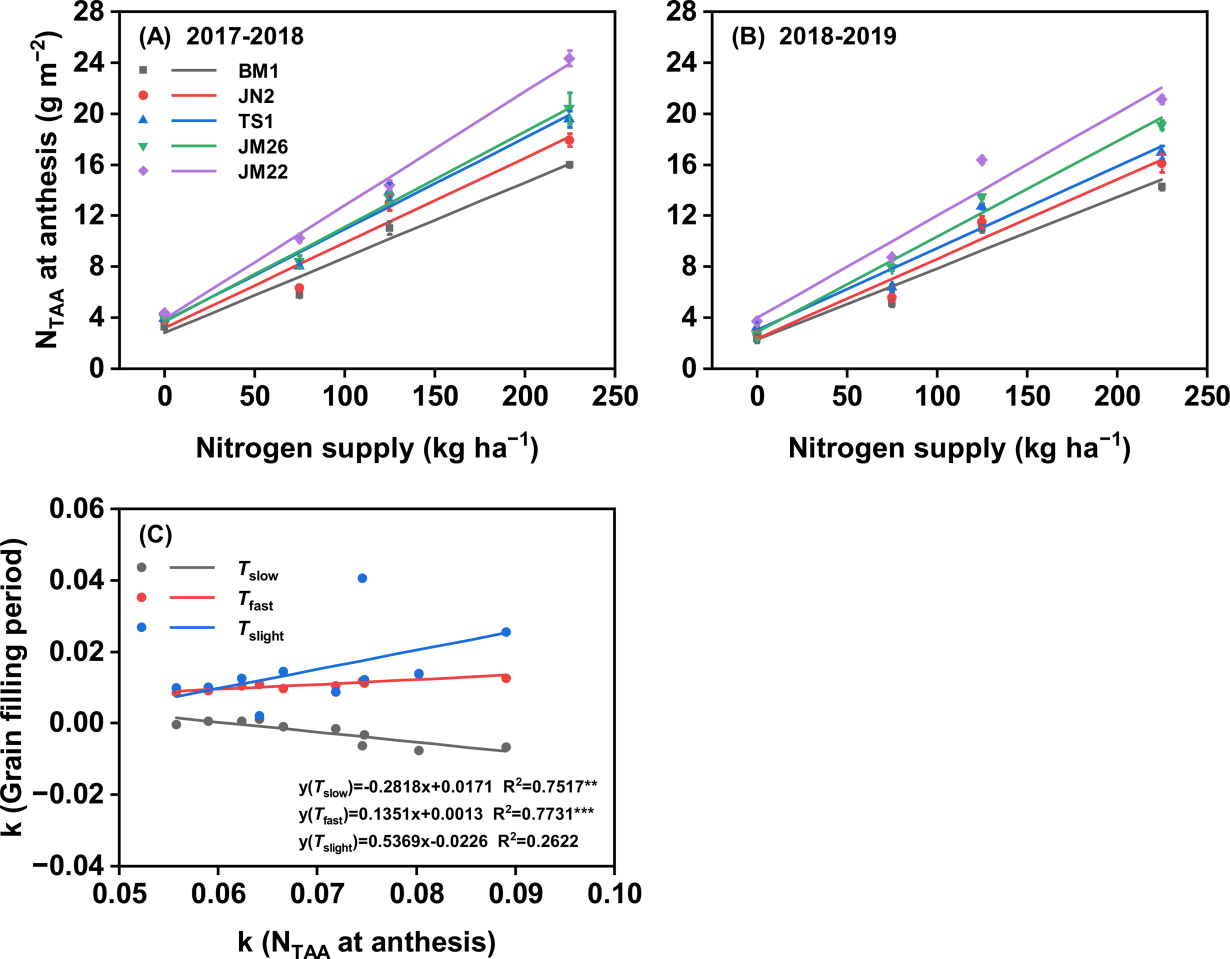
Effects of N supply on total N accumulation amount (N_TAA_) at anthesis among wheat cultivars in 2017-2018 **(A)** and 2018-2019 **(B)**. **(C)** the correlation between sensitivity of N_TAA_ at anthesis to N supply and grain filling period to N supply. k, sensitivity of grain filling period and N_TAA_ at anthesis to N supply; *T*_slow_, duration of slow-increase period; *T*_fast_, duration of fast-increase period; *T*_slight_, duration of slight-increase period. Values represented mean ± SE. *** indicated *P* < 0.001, ** indicated *P* < 0.01, * indicated *P* < 0.05, and ns indicated no significant difference.

The N_TAA_ at anthesis of five wheat cultivars increased along with the increasing rate of N supply in both growing seasons (**Figures 5A, B**). At the same N rate, JM22 consistently had a higher N_TAA_ at anthesis than the other cultivars, while BM1 had the lowest N_TAA_ at anthesis. The differences in N_TAA_ at anthesis between cultivars gradually widened when N application increased from 0 kg N ha^−1^ to 225 kg N ha^−1^, and the order of sensitivity of N_TAA_ at anthesis to N supply was JM22 > JM26 > TS1 > JN2 > BM1 (**Supplementary Table S4**). Strikingly, the sensitivity of N_TAA_ at anthesis to N supply was positively correlated with the sensitivity of *T*_fast_ to N supply (*P* < 0.001), but negatively correlated with the sensitivity of *T*_slow_ to N supply (*P* < 0.01), while independent of the sensitivity of *T*_slight_ to N supply (**Figure 5C**). Furthermore, the GW and *T*_fast_ were positively correlated to N_TAA_ at anthesis (*P* < 0.001), but did not correlate with anthesis biomass across the cultivars and N supplies (**Supplementary Figure S1**).

### 3.4 SPAD in response to N supply and the relationship between chlorophyll degradation rate and grain filling characteristics

**Figure 6:**
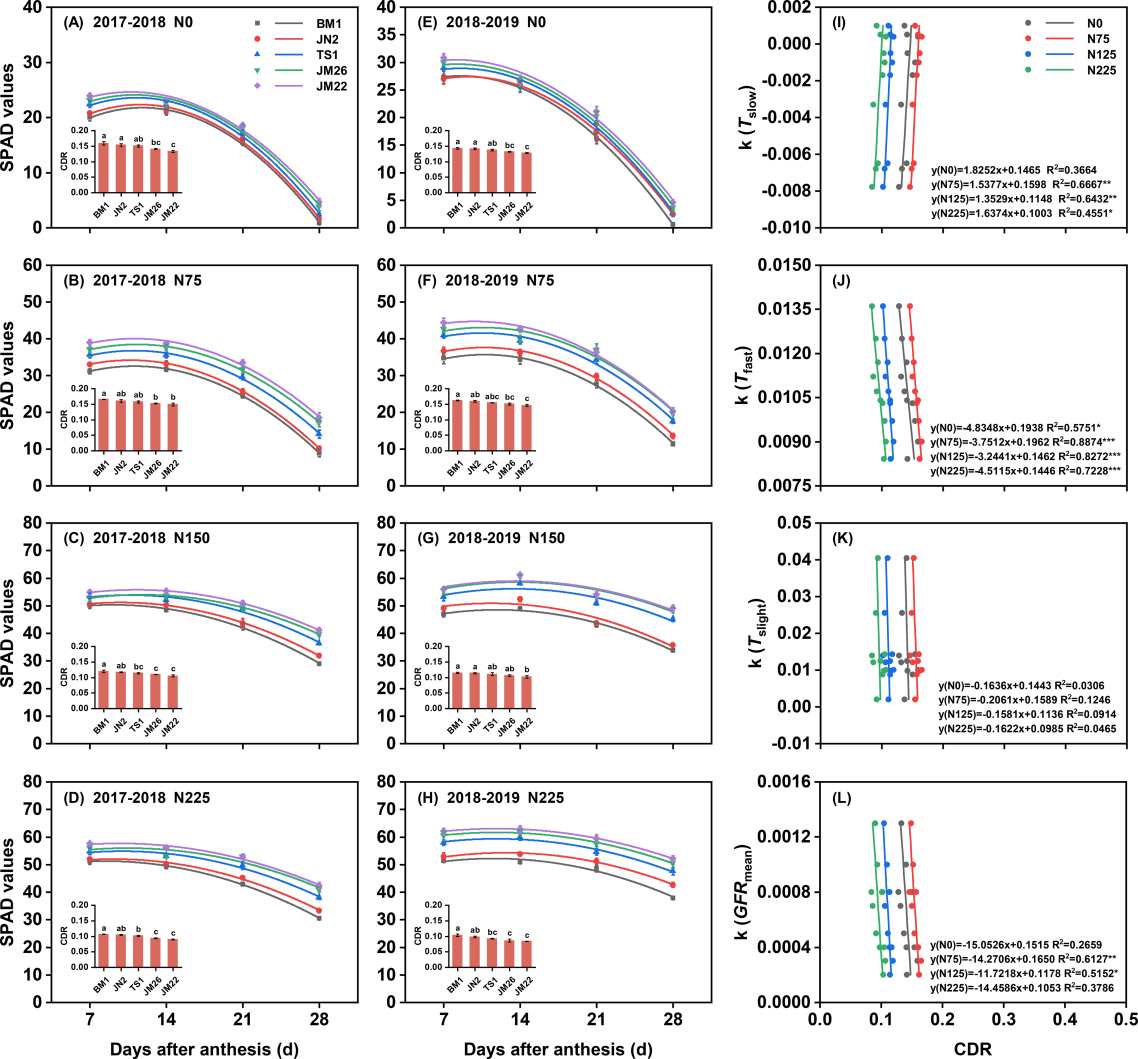
Effects of N supply on SPAD value and chlorophyll degradation rate (CDR) of flag leaves among wheat cultivars. Correlation between CDR and sensitivity of duration of slow-increase period (*T*_slow_) to N supply **(I)**, duration of fast-increase period (*T*_fast_) to N supply **(J)**, duration of slight-increase period (*T*_slight_) to N supply **(K)**, and mean grain filling rate (*GFR*_mean_) to N supply **(L)**. **(A–D)** represented SPAD values and CDR under 0,75, 150, and 225 kg N ha^−1^ in 2017-2018, respectively. **(E–H)** represented SPAD values and CDR under 0,75, 150, and 225 kg N ha^−1^ in 2018-2019, respectively. k, sensitivity of grain filling parameters to N supply. Values represented mean ± SE, and different lowercase letters indicated significant differences between treatments at *P* < 0.05. *** indicated *P* < 0.001, ** indicated *P* < 0.01, * indicated *P* < 0.05, and ns indicated no significant difference.

SPAD values increased with higher N supply, and high N-responsiveness wheat cultivars maintained their dominance under equivalent N levels (**Figures 6A−H**). As growth progressed, SPAD values gradually declined, showing a rapid decrease under 0 and 75 kg N ha^−1^ treatments while remaining relatively high under 150 and 225 kg N ha^−1^. Conversely, increased N supply reduced the CDR of wheat cultivars in both growing seasons, with CDR consistently following the order: JM22< JM26< TS1< JN2< BM1. In 2017–2018 and 2018-2019, the CDR of JM22 was 2.2-16.5% and 2.8-19.1% lower than that of other cultivars, respectively. Additionally, CDR was negatively correlated with the sensitivity of *T*_fast_ to N supply but not with the sensitivity of *T*_slight_ to N supply at different N supplies (**Figures 6J, K**). Under 75 and 125 kg N ha^−1^, CDR correlated positively with the sensitivity of *T*_slow_ to N supply and negatively with the sensitivity of *GFR*_mean_ to N supply (**Figures 6I, L**, **Supplementary Figure S2**).

### 3.5 N translocation, N use efficiency and N nutrient index in response to N supply

**Figure 7:**
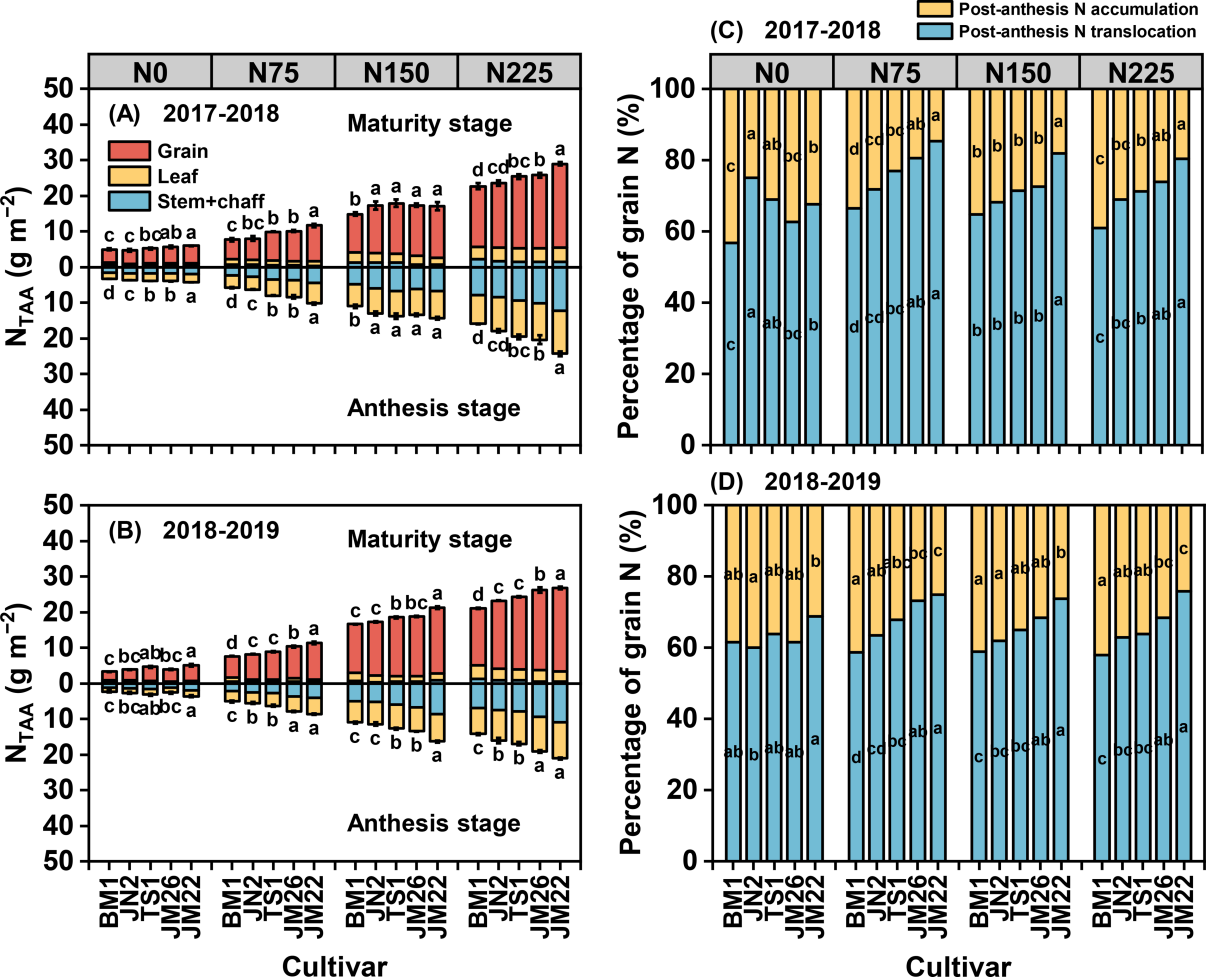
Effects of N supply on total N accumulation amount (N_TAA_) (**A**, 2017-2018; **B**, 2018-2019) and grain N origin (**C**, 2017-2018; D, 2018-2019) of five wheat cultivars. Values represented mean ± SE, and different lowercase letters indicated significant differences between treatments at *P* < 0.05.

N remobilization contributed differentially among cultivars, JM22 derived 70-80% of grain N from pre-anthesis biomass, compared to 60% for BM1. The remaining N originated from post-anthesis uptake, with stems + chaff contributing more than leaves to remobilized N (**Figures 7A−D**, **Supplementary Table S5**). At the same N rate, JM22 had higher NUtE, NUpE, NTE, and CNTA, which in turn contributed to higher NUE (**Supplementary Table S5**). Under 75, 150, and 225 kg N ha^−1^, NUE of JM22 increased by 14.5-63.9%, 25.4-80.6% and, 9.9-84.0% in 2017-2018, and by 10.1-42.4%, 15.2-67.3%, and 14.9-69.0% in 2018-2019, respectively, compared to other wheat cultivars. In addition, when the application rates ≤150 kg N ha^−1^, only JM22 approached optimal N status (NNI ≈ 1.0), while all other cultivars maintained suboptimal NNI values (< 1.0) (**Supplementary Figure S3**). These results demonstrated that the critical N requirement for optimal vegetative growth in JM22 closely matched the calculated N_cs_ values (147.40 kg N ha^−1^ in 2017-2018, 152.02 kg N ha^−1^ in 2018-2019).

### 3.6 N translocation amount in response to N supply and its relationship to grain filling period and chlorophyll degradation rate

**Figure 8:**
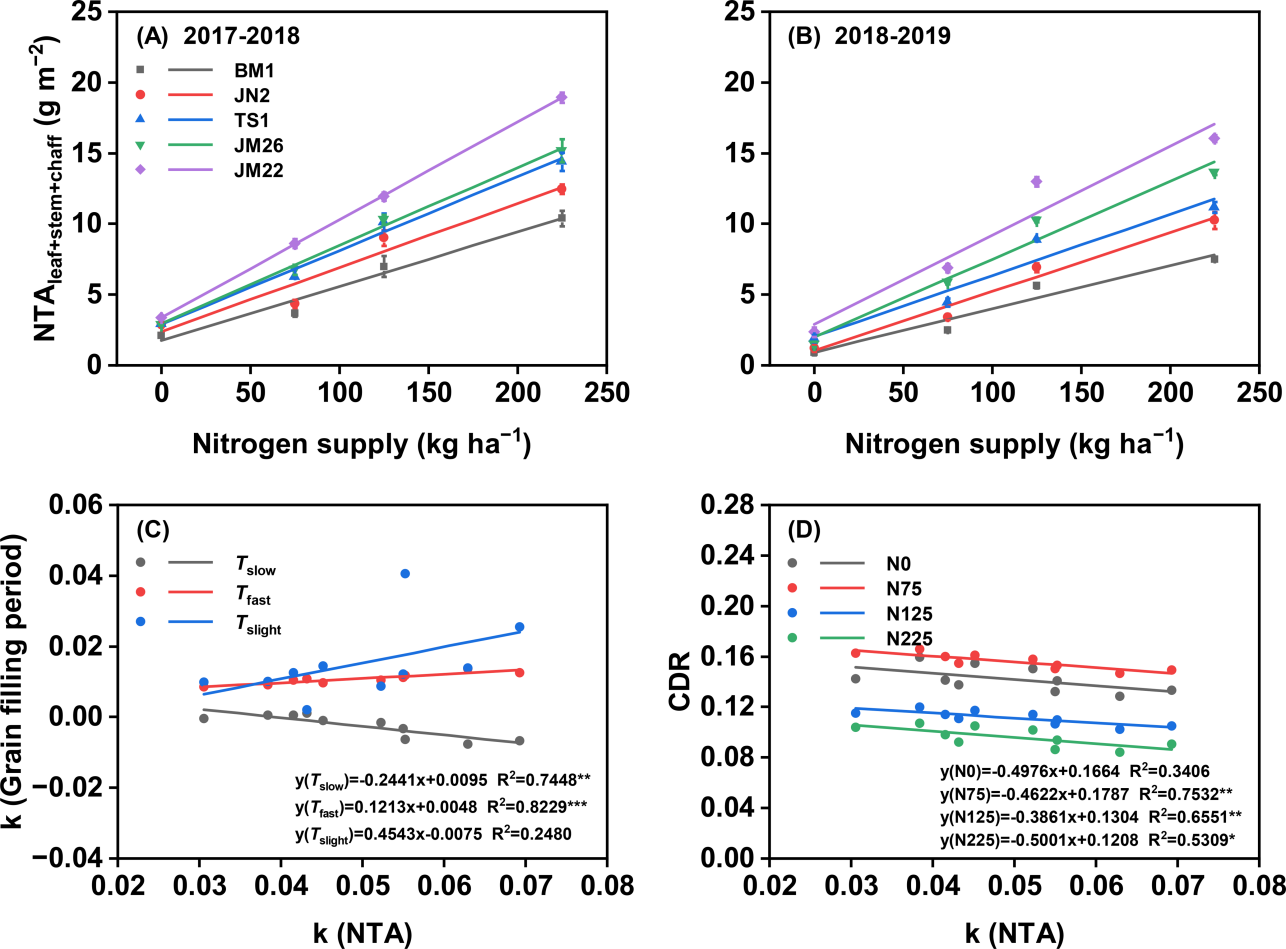
Effects of N supply on N translocation amount (NTA_leaf+stem+chaff_) in five wheat cultivars in 2017-2018 **(A)** and 2018-2019 **(B)**. Correlation between sensitivity of NTA to N supply and sensitivity of grain filling period to N supply **(C)** and chlorophyll degradation rate (CDR) **(D)**. k, sensitivity of grain filling period and NTA to N supply; *T*_slow_, duration of slow-increase period; *T*_fast_, duration of fast-increase period; *T*_slight_, duration of slight-increase period. Values represented mean ± SE. *** indicated *P* < 0.001, ** indicated *P* < 0.01, * indicated *P* < 0.05, and ns indicated no significant difference.

NTA from vegetative tissues (leaf + stem + chaff) showed a significant positive response to N application rates in both growing seasons. Furthermore, cultivar differences in NTA became increasingly distinct at higher N supply levels (**Figures 8A, B**). NTA and the sensitivity of NTA to N supply was consistently higher in JM22 than in the other cultivars and the lowest in BM1 at the same N rate (**Supplementary Table S6**). The sensitivity of NTA to N supply correlated negatively with the sensitivity of *T*_slow_ to N supply (*P* < 0.01), positively correlated with the sensitivity of *T*_fast_ to N supply (*P* < 0.001), and unrelated to the sensitivity of *T*_slight_ to N supply (**Figure 8C**). Similarly, the sensitivity of NTA to N supply correlated negatively (*P* < 0.001) with CDR at 75 (*P* < 0.01), 125 (*P* < 0.01), and 225 (*P* < 0.05) kg N ha^−1^, but not at 0 kg N ha^−1^ (**Figure 8D**).

### 3.7 Relationship between grain filling characteristics and other parameters

**Figure 9:**
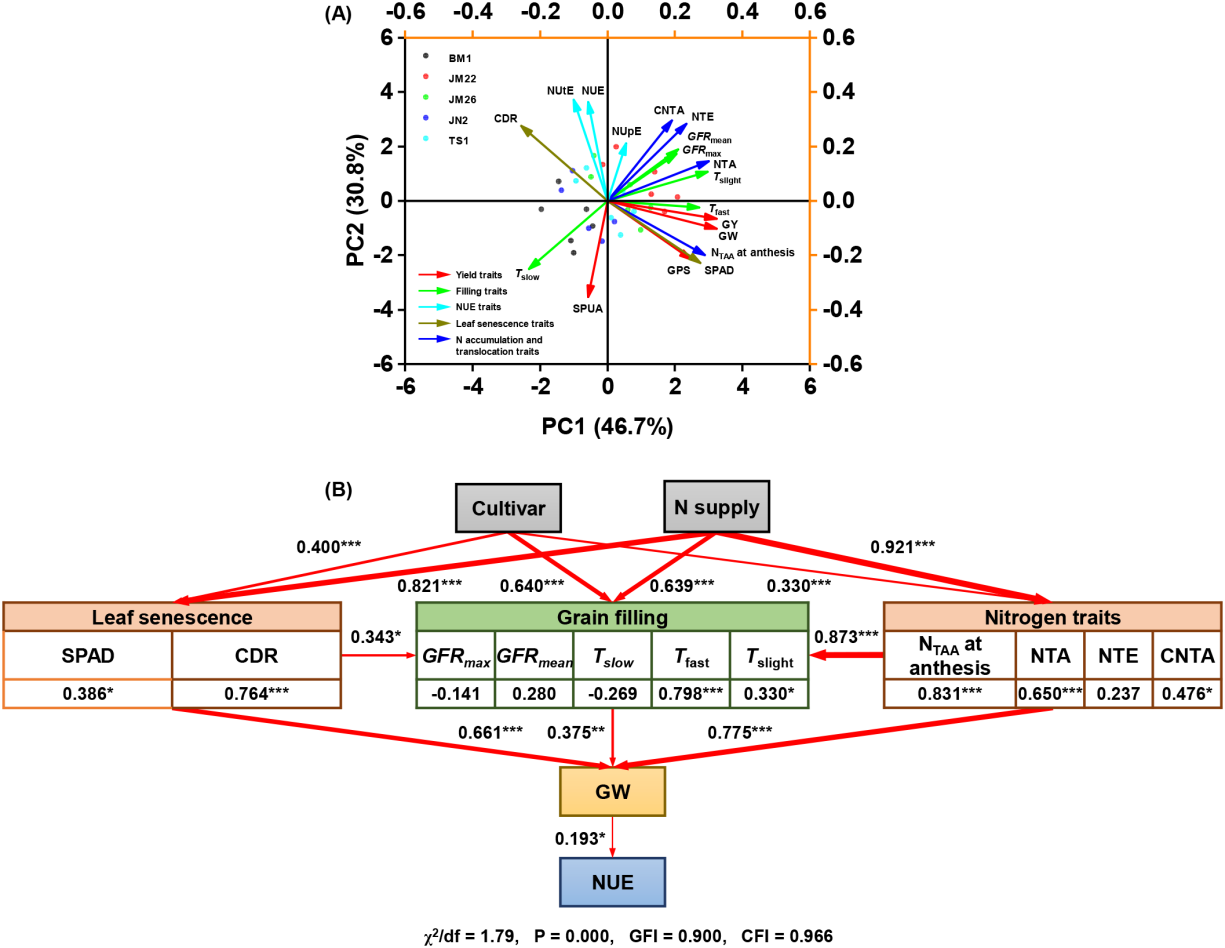
Relationships between yield traits, grain filling traits, and N-related traits. **(A)** Principal component analysis (PCA) revealed the linkages of yield traits with grain filling traits and N-related traits. **(B)** Structural equation modeling (SEM) illustrated the direct or indirect effects of cultivar, N supply, N-related traits, and duration of fast-increase period (*T*_fast_) on wheat grain weight and NUE. Red lines indicate positive correlations. Arrow width corresponds to the magnitude of statistically significant standardized path coefficients, with thicker lines representing stronger relationships. GY, grain yield; GW, grain weight; GPS, grains per spike; SPUA, spikes per unit area; *GFR*_max_, maximum grain filling rate; *GFR*_mean_, mean grain filling rate; *T*_slow_, duration of slow-increase period; *T*_fast_, duration of fast-increase period; *T*_slight_, duration of slight-increase period; NUtE, N utilization efficiency; NUpE, N uptake efficiency; NUE, N use efficiency; NTA, N translocation amount; NTE, N translocation efficiency; CNTA, contribution rate of N translocation amount to grain; N_TAA_, total N accumulation amount; CDR, chlorophyll degradation rate. *** indicated *P* < 0.001, ** indicated *P* < 0.01, * indicated *P* < 0.05, and ns indicated no significant difference.

According to the PCA, the first two principal components together explained 77.5% of the variation in yield traits. GY was positively correlated to GW and GPS and negatively correlated to SPUA. SPAD, N_TAA_ at anthesis, and *T*_ast_ were the most important factors affecting GW, whereas NUtE was the most important factor affecting NUE (**Figure 9A**). The SEM revealed that cultivar and N supply increased GW by inducing leaf senescence, grain filling, N accumulation, and N translocation (**Figure 9B**). With increased N_TAA_ at anthesis and lower CDR, *T*_fast_ was driven to prolong, which resulted directly in wheat GW and NUE improvement, and the contribution of N_TAA_ at anthesis to *T*_fast_ was higher than that of CDR. Additionally, *T*_fast_ extension also indirectly increased GW and NUE by promoting increased post-anthesis N translocation.

## 4 Discussion

The consistent rise in both absolute yield and yield increments across release years provides strong evidence that contemporary wheat breeding prioritizes and achieves enhanced N responsiveness, i.e., high N efficiency (**Figure 2**, **Supplementary Figure S1**). Meanwhile, the GY_max_ and N_cs_ increased along with the increase of N responsiveness, aligning with established findings in wheat and maize (Liu et al., 2023; Peng et al., 2022). Additionally, yield segregation became increasingly significant with N supply, with the order of GY was JM22 > JM26 > TS1 > JN2 > BM1 (**Supplementary Figure S1**), suggesting that N contributes a wider range of yield enhancement in high-yielding wheats, which may be attributed to higher N demand and NUE (Barraclough et al., 2014; Islam et al., 2021; Peng et al., 2022; Ren et al., 2023; Tian et al., 2016).

### 4.1 N responsiveness was related to grain filling process

The yield potential of wheat was contributed by GW, SPUA, and GPS, and GW and GPS contribute to the increase of yield in the appropriate range of SPUA (Akin et al., 2017; Ma et al., 2022). In this study, GPS was the primary contributor to yield among these factors. In addition, GW also explained 85% of the variation in GY, and the direct path coefficient of GW on GY was 0.478 (*P* < 0.001, **Figure 2F**). The significant cultivar × N interaction for GY and GW (*P* < 0.05, **Supplementary Table S1**) suggests that the N-mediated increase in GY may also be driven by the enhancement of GW among different genotypes in addition to GPS. Numerous studies have concluded that the formation of GW in wheat is regulated by the grain filling process and directly determines the yield, whereas *GFR* and effective *GFD* are considered to be the parameters that characterize the filling grains (Ma et al., 2022; Zhang et al., 2022). As demonstrated in our study and supported by previous research (He et al., 2020), enhanced *GFR* coupled with prolonged effective *GFD* significantly increases grain storage capacity (**Supplementary Table S1**).

Across cultivar and N supply combinations, the present study demonstrated the role of grain filling traits in N responsiveness based on significant positive correlations between grain filling parameters (*GFR*_max_, *GFR*_mean_, *T*_fast_, and *T*_slight_) and GW (**Figure 3**, **Supplementary Figure S1B**). In fact, *T*_fast_ explained about 74% of the variation in GW, and RFM analysis indicated that *T*_fast_ (*P <* 0.001) had the most significant importance for GW establishment (**Figure 4H**, **Supplementary Figure S1A**), suggesting that the high responsiveness of wheat GW to N supply may be explained mainly by *T*_fast_ in the grain filling trait, which provides new insights into genotypic differences in N responsiveness in wheat at the filling level. Moreover, the effective grain filling period is determined by *T*_slow_, *T*_fast_, and *T*_slight_ (Ma et al., 2022; Zhang et al., 2022). Research has established that the duration of grain filling periods (*T*_slow_, *T*_fast_, and *T*_slight_) is modulated by both environmental and genetic factors (Hu et al., 2023; Liu et al., 2016). Consistently, this study revealed that *T*_fast_ and *T*_slight_ were more sensitive to increased N supply and cultivar replacement, while Pearson correlation coefficients also identified the *T*_fast_ as the main driving factors that mediated N responsiveness (**Figure 4G**), which explained the genetic differences in the response of N responsiveness to N supply regulated mainly by the *T*_fast_. This might provide new targets for the diagnosis and high-yield breeding of N-efficient wheat cultivars in the future. Overall, the high N-responsiveness cultivar JM22 increased *T*_fast_, *GFR*_max_, and *GFR*_mean_ compared to other cultivars, particularly under elevated N conditions (**Figure 4**, **Supplementary Table S2**). These physiological advantages extended both the duration and efficiency of photoassimilate translocation to developing grains. Under high N supply treatment, the *T*_fast_ and *T*_slight_ appeared earlier and lasted longer in JM22 than in other cultivars, resulting in a longer effective grain filling period. This temporal expansion of *T*_fast_ and *T*_slight_ significantly enhanced dry matter accumulation, ultimately contributing to greater final GW, a phenomenon well-documented in recent studies (Ma et al., 2022; Zhang et al., 2022).

### 4.2 Adequate anthesis N sources and lower CDR are key to increasing *T*_fast_

Grain filling duration may be limited by source capacity (Rivera-Amado et al., 2020). Pre-anthesis plant N sink capacity influences canopy development (Yin et al., 2019) and photosynthetic intensity (Gutiérrez et al., 2013; Wang et al., 2016), and may affect grain filling traits. Recent research suggests that improved grain filling capacity may primarily result from enhanced N accumulation at both individual plant and population levels (Bancal, 2009; Martínez-Peña et al., 2022). Our results demonstrated that N_TAA_ at anthesis increased linearly with both N supply level and cultivar N responsiveness. Notably, the sensitivity of N_TAA_ at anthesis to N supply was positively correlated with the sensitivity of *T*_fast_ to N supply (*P* < 0.001), and negatively correlated with the sensitivity of *T*_slow_ to N supply (*P <* 0.01), and not correlated with the sensitivity of *T*_slight_ to N supply (**Figures 5A−C**). These results indicate that an increase in N_TAA_ at anthesis is the key driver of the enhanced *T*_fast_ across different combinations of cultivar and N supply, i.e., longer *T*_fast_ gains in high N-responsiveness wheat cultivars are achieved through increased N sources at anthesis. The study of Bancal (2009) also pointed out that grain filling traits seem to be determined by the source (stem-stored N at anthesis and post-anthesis N uptake).

N-mediated regulation of *GFR* and *GFD* appears to be functionally linked to photosynthetic activity maintenance (He et al., 2020; Makino, 2011). Previous studies have demonstrated that leaf senescence rate was negatively correlated with *GFD*, and increasing leaf greening time was beneficial for prolonging *GFD* (Liu et al., 2016; Luo et al., 2018; Wu et al., 2018). Consistent with these findings, our data revealed that the CDR under different N supplies was positively correlated with the sensitivity of *T*_slow_ to N supply and negatively correlated with the sensitivity of *T*_fast_ and *GFR*_mean_ to N supply (**Figure 6**). Meanwhile, the higher leaf N levels of the high-N-responsiveness wheat cultivars explained their lower CDR (**Figures 6**, **7**). In other words, higher N-responsiveness wheat cultivars still maintained longer leaf greening time, larger photosynthetic area, and longer photosynthetic duration during the filling period, allowing more assimilates to be transferred to the grain, which could significantly increase *T*_fast_ and *GFR*_mean_ (Ma et al., 2022; Wang et al., 1999).

Moreover, we noted that the two-year field experiment encompassed contrasting inter-annual climatic conditions, particularly in precipitation patterns, with growing-season rainfall measuring 346.4 mm in 2017–2018 and 689.4 mm in 2018-2019 (**Figure 1**). This variability provided a robust opportunity to test the consistency of the observed relationships under different environmental stresses. Notably, while grain yields and *T*_fast_ varied between years−being generally lower in the drier year−the fundamental relationships driving N responsiveness remained unchanged. Specifically: (1) The hierarchy of grain filling parameters, with *T*_fast_ being the primary driver of GW variation, was conserved across both years. (2) The positive correlation between pre-anthesis N accumulation and *T*_fast_ duration, and its subsequent effect on final GW, held true regardless of precipitation level. (3) The cultivars identified as high-N-responsiveness maintained their relative superiority across N levels in both seasons. The consistency of these core findings suggests that the mechanism we propose−whereby adequate pre-anthesis N accumulation promotes *T*_fast_ duration and enhances N responsiveness−is a robust physiological pathway that operates under varying water availability. The inter-annual differences primarily modulated the magnitude of the response rather than the direction or relative importance of the underlying traits. This reinforces the conclusion that targeting *T*_fast_ and pre-anthesis N management is a viable strategy for improving NUE across a range of representative field conditions.

### 4.3 *T*_fast_ induces N translocation to enhance NUE

High N-responsiveness wheat cultivars achieve superior NUE and yield through dual optimization of pre-anthesis N accumulation and post-anthesis N translocation (Islam et al., 2021; Koutroubas et al., 2012; Lian et al., 2023). Previous studies have reported that increased N translocation to the grain can be induced by higher N accumulation before anthesis (Pang et al., 2014; Tian et al., 2016). Our findings indicated that genetic variation in pre-anthesis N uptake mediated variation in NUE in response to N supply (**Figure 7**, **Supplementary Table S5**). Higher N-responsiveness wheat cultivars uptake and accumulated more N at pre-anthesis, especially under high N supply (**Figure 7**), which increased NUE and also provided a material basis for post-anthesis N translocation to grains, which may be attributed to the increased N uptake and assimilation capacity during this growth period (Bancal, 2009; Barraclough et al., 2010). Our monitoring results also demonstrated the point that the highest NUpE was observed for the highly N-responsiveness wheat cultivar JM22 than other cultivars (**Supplementary Table S5**), as NUpE is defined as a function of N uptake per unit of N supply (Peng et al., 2022). A recent study found that higher N uptake at jointing stage was the main reason for mediating N accumulation amount (Peng et al., 2022; Tian et al., 2016). Therefore, genetic breeding efforts for high-NUE wheat should prioritize enhancing pre-anthesis N uptake over post-anthesis (Zhou et al., 2018). This finding is consistent with our results.

N availability after anthesis largely determines GW in wheat (Barraclough et al., 2014; Przulj and Momcilovic, 2001). Wheat grain N mainly originates from the remobilization of N stored in vegetative organs (about 70-80% of grain N) at anthesis (**Figures 7C, D**), and a fraction from post-anthesis N uptake (Barraclough et al., 2010; Xu et al., 2012, 2005). Analysis of N translocation patterns revealed significant genotypic variation among wheat cultivars with differing N responsiveness during grain filling. High N-responsiveness cultivars demonstrated enhanced N remobilization from vegetative tissues to developing grains. Notably, stems + chaff exhibited greater translocation efficiency compared to leaves (**Supplementary Table S5**). This suggests that stems + chaff is also one of the indispensable N sinks in addition to leaves (Przulj and Momcilovic, 2001; Wang et al., 2016). Remobilization of N from leaves to grains is responsible for inducing leaf senescence, whereas N stored in stems + chaff is remobilized in preference to photosynthetic N in leaves would help to prolong the leaf stay-green period and hence increasing photosynthetic capacity (Gutiérrez et al., 2013; Makino, 2011; Yin et al., 2019). Regression analysis indicated that the sensitivity of NTA to N supply was positively correlated with the sensitivity of *T*_fast_ to N supply and negatively correlated with CDR (**Figures 8C, D**). According to PCA and SEM, NUtE which is the most dominant factor affecting NUE rather than NUpE, and high N-responsiveness wheat cultivars increase NUE under N supply by directly mediating increased N accumulation at anthesis, and also indirectly by increasing *T*_fast_-induced increases in post-anthesis N translocation (**Figures 9A, B**). These results emphasize that increasing *T*_fast_ and N translocation over a temporal distribution is essential for GW establishment (Li et al., 2020; Wu et al., 2018; Yan et al., 2019; Zhang et al., 2022), that enhanced post-anthesis N translocation rather than N uptake is the main reason for increased NUE, and that high NUE ensures higher N responsiveness (Xu et al., 2024). Overall, higher NUE implies lower N fertilizer inputs and higher economic outputs (Hu et al., 2023), as higher yields can still be obtained at 0 kg N ha^−1^ (**Figures 2A, B**).

In summary, high wheat grain yield responsiveness to N involves a sequential mechanism: it is driven by enhanced pre-anthesis N assimilation, which results in prolonged *T*_fast_. Prolongation of *T*_fast_ increased N translocation after anthesis and ultimately increased the efficiency of N translocation to grains.

## 5 Conclusion

The high N responsiveness of grain yield in wheat is contributed by the sensitivity of *T*_fast_ to N supply, which can be achieved by adequate pre-anthesis N accumulation and lower CDR. Specifically, sufficient N sources at anthesis ensure that plants have the necessary resources to support grain development, while lower CDRs help maintain NUE during the critical growth stages. Moreover, prolonging *T*_fast_ increased post-anthesis N translocation capacity in wheat, and this increased capacity contributes to improved NUE by allowing more efficient remobilization of N from vegetative tissues to developing grains. Therefore, high N accumulation at anthesis and longer *T*_fast_ may be a potential target for diagnosing N efficiency in wheat cultivation in the future. By selecting for these traits, breeders can develop wheat cultivars that are more efficient in NUE, which is paramount in maximizing resource efficiency in sustainable agriculture.

## Data Availability

The original contributions presented in the study are included in the article/**Supplementary Material**. Further inquiries can be directed to the corresponding author.
