# Supplementary material for: High nitrogen use efficiency in wheat is explained by a longer fast-increase period and adequate pre-anthesis nitrogen accumulation
Source: Front Plant Sci. 2026 Jan 23;16:1727679. doi: 10.3389/fpls.2025.1727679 (PMC12877789; doi:10.3389/fpls.2025.1727679)
Supplement: Supplementary file 1 [file DataSheet1.docx]

**Supplementary material**

**TABLE S1** Effects of N supply on yield and yield components of five wheat cultivars.

| Treatment | SPUA | GPS | GW (g 1000-grain^−1^) | GY (t ha^−1^) |
| --- | --- | --- | --- | --- |
| Year |  |  |  |  |
| 2017-2018 | 665 b | 20 b | 39.8 a | 4.3 b |
| 2018-2019 | 809 a | 23 a | 37.6 b | 4.5 a |
| N supply |  |  |  |  |
| 0 | 718 b | 9 d | 34.9 c | 2.0 d |
| 75 | 744 a | 17 c | 38.5 b | 4.1 c |
| 150 | 737 ab | 29 b | 40.5 a | 5.7 b |
| 225 | 747 a | 32 a | 40.8 a | 5.9 a |
| Cultivar |  |  |  |  |
| BM1 | 798 a | 16 e | 36.4 e | 3.3 e |
| JN2 | 770 b | 20 d | 37.7 d | 3.9 d |
| TS1 | 755 b | 23 c | 38.7 c | 4.4 c |
| JM26 | 708 c | 24 b | 39.4 b | 4.9 b |
| JM22 | 652 d | 26 a | 41.1 a | 5.6 a |
| ANOVA |  |  |  |  |
| Y | *** | *** | *** | *** |
| N | ns | *** | *** | *** |
| C | *** | *** | *** | *** |
| Y * N | ** | *** | *** | *** |
| Y * C | *** | *** | *** | *** |
| N * C | *** | *** | *** | *** |
| Y * N * C | ns | ns | *** | ns |

Note: Y, year; N, nitrogen supply; C, cultivar; SPUA, spikes per unit area. GPS, grains per spike; GW, grain weight; GY, grain yield. Values represented mean, and different lowercase letters indicated significant differences between treatments at *P* < 0.05. *** indicated *P* < 0.001, ** indicated *P* < 0.01, * indicated *P* < 0.05, and ns indicated no significant difference.

**TABLE S2** Effects of N supply on grain filling parameters of five wheat cultivars.

| Year | Nitrogen supply  (kg ha^−1^) | Cultivar | *GFR*_max_  (mg grain^−1^ d^−1^) | *GFR*_mean_  (mg grain^−1^ d^−1^) | *T*_slow_  (d) | *T*_fast_  (d) | *T*_slight_  (d) |
| --- | --- | --- | --- | --- | --- | --- | --- |
| 2017-2018 | 0 | BM1 | 2.07 d | 1.44 a | 11.5 a | 8.8 e | 11.0 d |
|  |  | JN2 | 2.15 cd | 1.44 a | 11.0 b | 9.8 d | 12.7 c |
|  |  | TS1 | 2.17 bc | 1.41 b | 10.4 c | 10.4 c | 16.1 b |
|  |  | JM26 | 2.18 b | 1.43 a | 10.4 c | 10.7 b | 13.0 c |
|  |  | JM22 | 2.19 a | 1.39 c | 10.3 c | 12.5 a | 18.8 a |
|  | 70 | BM1 | 2.12 c | 1.45 b | 11.3 a | 9.6 d | 11.3 d |
|  |  | JN2 | 2.18 c | 1.51 a | 10.8 b | 10.2 c | 13.2 c |
|  |  | TS1 | 2.20 b | 1.44 b | 10.3 d | 12.0 a | 15.6 b |
|  |  | JM26 | 2.22 b | 1.46 b | 10.5 c | 11.3 b | 17.3 a |
|  |  | JM22 | 2.26 a | 1.45 b | 9.2 d | 12.8 a | 20.8 a |
|  | 150 | BM1 | 2.29 c | 1.49 c | 11.6 a | 10.4 d | 12.8 b |
|  |  | JN2 | 2.43 b | 1.62 b | 10.9 b | 11.2 c | 14.1 b |
|  |  | TS1 | 2.42 b | 1.63 b | 10.2 b | 12.6 b | 16.7 a |
|  |  | JM26 | 2.45 b | 1.52 b | 9.8 c | 11.7 c | 19.8 a |
|  |  | JM22 | 2.55 a | 1.71 a | 9.1 d | 13.8 a | 21.8 a |
|  | 225 | BM1 | 2.15 d | 1.51 c | 11.5 a | 10.8 d | 13.0 c |
|  |  | JN2 | 2.24 c | 1.50 c | 10.7 b | 11.9 d | 16.0 c |
|  |  | TS1 | 2.30 b | 1.54 b | 10.0 b | 12.8 c | 17.9 c |
|  |  | JM26 | 2.26 c | 1.65 a | 9.0 c | 13.5 b | 22.4 b |
|  |  | JM22 | 2.35 a | 1.62 a | 8.6 c | 15.3 a | 24.9 a |
| 2018-2019 | 0 | BM1 | 1.84 c | 1.22 d | 14.0 a | 9.7 d | 11.9 c |
|  |  | JN2 | 1.86 bc | 1.24 d | 13.4 a | 10.4 c | 12.9 c |
|  |  | TS1 | 1.92 b | 1.29 c | 12.2 b | 11.4 b | 15.9 b |
|  |  | JM26 | 2.00 a | 1.32 b | 12.1 b | 12.0 ab | 16.6 ab |
|  |  | JM22 | 2.06 a | 1.37 a | 12.4 b | 12.5 a | 17.4 a |
|  | 70 | BM1 | 2.12 ab | 1.42 ab | 13.5 a | 10.7 c | 12.5 b |
|  |  | JN2 | 2.14 ab | 1.43 ab | 12.4 a | 11.8 bc | 14.4 b |
|  |  | TS1 | 2.06 b | 1.38 b | 11.9 a | 12.7 ab | 16.0 b |
|  |  | JM26 | 2.13 ab | 1.44 ab | 11.6 b | 13.6 a | 18.6 a |
|  |  | JM22 | 2.22 a | 1.50 a | 11.4 b | 13.9 a | 18.9 a |
|  | 150 | BM1 | 2.01 c | 1.27 c | 13.7 a | 11.4 b | 14.2 b |
|  |  | JN2 | 2.01 c | 1.35 c | 12.7 b | 12.5 a | 16.3 ab |
|  |  | TS1 | 2.05 c | 1.37 c | 12.0 b | 13.6 a | 15.7 ab |
|  |  | JM26 | 2.18 b | 1.45 b | 11.5 c | 13.7 a | 19.1 a |
|  |  | JM22 | 2.39 a | 1.60 a | 11.2 c | 14.9 a | 19.5 a |
|  | 225 | BM1 | 1.91 d | 1.33 d | 13.8 a | 11.6 c | 13.8 c |
|  |  | JN2 | 2.08 c | 1.37 c | 13.4 ab | 12.8 c | 15.4 c |
|  |  | TS1 | 2.15 b | 1.41 bc | 12.4 b | 13.8 bc | 16.5 b |
|  |  | JM26 | 2.18 b | 1.49 b | 11.3 c | 14.8 b | 19.5 ab |
|  |  | JM22 | 2.32 a | 1.54 a | 10.5 d | 15.6 a | 20.7 a |
| Y |  |  | *** | *** | *** | *** | ns |
| N |  |  | *** | *** | *** | *** | *** |
| C |  |  | *** | *** | *** | *** | *** |
| Y * N |  |  | *** | *** | *** | *** | *** |
| Y * C |  |  | *** | *** | ns | *** | *** |
| N * C |  |  | *** | *** | *** | *** | *** |
| Y * N * C |  |  | *** | *** | *** | * | *** |

Note: Y, year; N, nitrogen supply; C, cultivar; *GFR*_max_, maximum grain filling rates; *GFR*_mean_, mean grain filling rates; *T*_slow_, duration of slow-increase period; *T*_fast_, duration of fast-increase period; *T*_slight_, duration of slight-increase period. Values represented mean, and different lowercase letters indicated significant differences between treatments at *P* < 0.05. *** indicated *P* < 0.001, ** indicated *P* < 0.01, * indicated *P* < 0.05, and ns indicated no significant difference.

**TABLE S3** Models and parameters describing the sensitivity of filling period to N supply in five wheat cultivars in 2017-2018 and 2018-2019.

| Year | Parameter | Cultivar | Model | R^2^ | k |
| --- | --- | --- | --- | --- | --- |
| 2017-2018 | *T*_slow_ | BM1 | y = 0.0004x + 11.4277 | 0.1085 | 0.0004 |
|  |  | JN2 | y = -0.0010x + 10.9661 | 0.6207 | -0.0010 |
|  |  | TS1 | y = -0.0017x + 10.4166 | 0.9671 | -0.0017 |
|  |  | JM26 | y = -0.0065x + 10.6530 | 0.8555 | -0.0065 |
|  |  | JM22 | y = -0.0068x + 10.0587 | 0.8614 | -0.0068 |
|  | *T*_fast_ | BM1 | y = 0.0090x + 8.8994 | 0.9775 | 0.0090 |
|  |  | JN2 | y = 0.0097x + 9.6959 | 0.9838 | 0.0097 |
|  |  | TS1 | y = 0.0103x + 10.8100 | 0.8436 | 0.0103 |
|  |  | JM26 | y = 0.0117x + 10.5029 | 0.8891 | 0.0117 |
|  |  | JM22 | y = 0.0125x + 12.2093 | 0.9323 | 0.0125 |
|  | *T*_slight_ | BM1 | y = 0.0100x + 10.9137 | 0.9099 | 0.0100 |
|  |  | JN2 | y = 0.0143x + 12.4045 | 0.9252 | 0.0143 |
|  |  | TS1 | y = 0.0087x + 15.6051 | 0.7354 | 0.0087 |
|  |  | JM26 | y = 0.0405x + 13.6180 | 0.9746 | 0.0405 |
|  |  | JM22 | y = 0.0255x + 18.7366 | 0.9566 | 0.0255 |
| 2018-2019 | *T*_slow_ | BM1 | y = -0.0005x + 13.8036 | 0.0510 | -0.0005 |
|  |  | JN2 | y = 0.0005x + 12.9199 | 0.0091 | 0.0005 |
|  |  | TS1 | y = 0.0010x + 12.0182 | 0.1782 | 0.0010 |
|  |  | JM26 | y = -0.0033x + 11.9902 | 0.8845 | -0.0033 |
|  |  | JM22 | y = -0.0078x + 12.2386 | 0.9301 | -0.0078 |
|  | *T*_fast_ | BM1 | y = 0.0084x + 9.9109 | 0.9196 | 0.0084 |
|  |  | JN2 | y = 0.0104x + 10.7179 | 0.9001 | 0.0104 |
|  |  | TS1 | y = 0.0107x + 11.6867 | 0.9070 | 0.0107 |
|  |  | JM26 | y = 0.0112x + 12.2830 | 0.8914 | 0.0112 |
|  |  | JM22 | y = 0.0136x + 12.7119 | 0.9720 | 0.0136 |
|  | *T*_slight_ | BM1 | y = 0.0098x + 12.0058 | 0.7883 | 0.0098 |
|  |  | JN2 | y = 0.0124x + 13.3681 | 0.6908 | 0.0124 |
|  |  | TS1 | y = 0.0020x + 15.8051 | 0.3207 | 0.0020 |
|  |  | JM26 | y = 0.0121x + 17.1071 | 0.8361 | 0.0121 |
|  |  | JM22 | y = 0.0139x + 17.5846 | 0.9681 | 0.0139 |

Note: *T*_slow_, duration of slow-increase period; *T*_fast_, duration of fast-increase period; *T*_slight_, duration of slight-increase period.

**TABLE S4** Models and parameters describing the sensitivity of total N accumulation amount (N_TAA_) to N supply at anthesis in five wheat cultivars in 2017-2018 and 2018-2019.

| Year | Parameter | Cultivar | Model | R^2^ | k |
| --- | --- | --- | --- | --- | --- |
| 2017-2018 | N_TAA_ at anthesis | BM1 | y = 0.0590x + 2.7529 | 0.9683 | 0.0590 |
|  |  | JN2 | y = 0.0666x + 3.1578 | 0.9509 | 0.0666 |
|  |  | TS1 | y = 0.0719x + 3.6955 | 0.9809 | 0.0719 |
|  |  | JM26 | y = 0.0746x + 3.6218 | 0.9933 | 0.0746 |
|  |  | JM22 | y = 0.0891x + 3.8517 | 0.9959 | 0.0891 |
| 2018-2019 | N_TAA_ at anthesis | BM1 | y = 0.0558x + 2.2490 | 0.9409 | 0.0558 |
|  |  | JN2 | y = 0.0624x + 2.3217 | 0.9614 | 0.0624 |
|  |  | TS1 | y = 0.0642x + 2.9810 | 0.9537 | 0.0642 |
|  |  | JM26 | y = 0.0748x + 2.8315 | 0.9849 | 0.0748 |
|  |  | JM22 | y = 0.0803x + 3.9283 | 0.9549 | 0.0803 |

**TABLE S5** Effects of N supply on N fertilizer utilization efficiencies of five wheat cultivars.

| Year | Nitrogen  supply  (kg ha^−1^) | Cultivar | NTA (g cm^−2^) | | NTE (%) | | CNTA (%) | | NUtE  (kg kg^−1^) | NUpE  (kg kg^−1^) | NUE  (kg kg^−1^) |
| --- | --- | --- | --- | --- | --- | --- | --- | --- | --- | --- | --- |
|  |  |  | Leaf | Stem+chaff | Leaf | Stem+chaff | Leaf | Stem+chaff |  |  |  |
| 2017- | 0 | BM1 | 0.92 c | 1.19 c | 0.24 c | 0.31 b | 0.25 c | 0.32 a |  |  |  |
| 2018 |  | JN2 | 1.44 b | 1.40 b | 0.35 a | 0.34 a | 0.38 a | 0.37 a |  |  |  |
|  |  | TS1 | 1.38 b | 1.51 b | 0.32 ab | 0.35 a | 0.33 ab | 0.36 a |  |  |  |
|  |  | JM26 | 1.36 b | 1.51 b | 0.30 b | 0.34 a | 0.30 bc | 0.33 a |  |  |  |
|  |  | JM22 | 1.61 a | 1.71 a | 0.32 ab | 0.34 a | 0.33 ab | 0.35 a |  |  |  |
|  | 75 | BM1 | 1.91 c | 1.74 c | 0.31 b | 0.28 b | 0.35 a | 0.33 a | 38.45 a | 1.08 c | 41.42 d |
|  |  | JN2 | 2.25 c | 2.10 c | 0.32 b | 0.30 b | 0.38 a | 0.35 a | 41.62 a | 1.17 c | 48.09 c |
|  |  | TS1 | 3.22 b | 3.02 b | 0.38 a | 0.35 a | 0.40 a | 0.37 a | 37.04 a | 1.40 b | 52.00 c |
|  |  | JM26 | 3.44 b | 3.32 b | 0.38 a | 0.37 a | 0.41 a | 0.40 a | 42.31 a | 1.40 b | 59.29 b |
|  |  | JM22 | 4.42 a | 4.18 a | 0.40 a | 0.38 a | 0.44 a | 0.42 a | 40.86 a | 1.66 a | 67.91 a |
|  | 150 | BM1 | 3.35 b | 3.63 d | 0.26 b | 0.29 c | 0.31 a | 0.34 c | 26.68 d | 1.02 b | 27.20 d |
|  |  | JN2 | 4.20 b | 4.81 c | 0.29 ab | 0.33 bc | 0.32 a | 0.36 bc | 25.33 d | 1.24 a | 31.38 c |
|  |  | TS1 | 4.55 ab | 5.55 b | 0.30 ab | 0.37 ab | 0.33 a | 0.40 ab | 29.24 c | 1.25 a | 36.36 b |
|  |  | JM26 | 4.81 ab | 5.49 b | 0.32 ab | 0.36 ab | 0.34 a | 0.39 abc | 32.59 b | 1.20 a | 39.16 b |
|  |  | JM22 | 5.83 a | 6.09 a | 0.36 a | 0.38 a | 0.40 a | 0.42 a | 40.44 a | 1.21 a | 49.11 a |
|  | 225 | BM1 | 4.64 b | 5.74 d | 0.26 a | 0.33 b | 0.27 a | 0.34 c | 17.18 c | 1.05 c | 18.01 e |
|  |  | JN2 | 5.64 b | 6.80 cd | 0.29 a | 0.35 b | 0.31 a | 0.38 bc | 19.50 c | 1.10 c | 21.45 d |
|  |  | TS1 | 6.32 ab | 8.08 bc | 0.30 a | 0.38 ab | 0.32 a | 0.41 abc | 21.92 b | 1.18 b | 25.90 c |
|  |  | JM26 | 6.42 ab | 8.73 b | 0.28 a | 0.38 ab | 0.31 a | 0.43 ab | 24.58 a | 1.23 b | 30.16 b |
|  |  | JM22 | 8.04 a | 10.89 a | 0.32 a | 0.43 a | 0.34 a | 0.46 a | 24.64 a | 1.35 a | 33.16 a |
| 2018- | 0 | BM1 | 0.41 c | 0.51 b | 0.16 d | 0.20 c | 0.17 d | 0.21 c |  |  |  |
| 2019 |  | JN2 | 0.57 bc | 0.62 b | 0.19 c | 0.21 c | 0.19 c | 0.21 c |  |  |  |
|  |  | TS1 | 0.91 ab | 0.97 ab | 0.25 b | 0.26 b | 0.23 b | 0.25 b |  |  |  |
|  |  | JM26 | 0.74 abc | 0.74 b | 0.25 b | 0.25 b | 0.22 b | 0.22 c |  |  |  |
|  |  | JM22 | 1.11 a | 1.22 a | 0.28 a | 0.30 a | 0.26 a | 0.29 a |  |  |  |
|  | 75 | BM1 | 1.21 e | 1.29 d | 0.22 d | 0.23 e | 0.20 d | 0.21 d | 36.82 c | 1.24 e | 45.80 d |
|  |  | JN2 | 1.69 c | 1.73 c | 0.27 c | 0.27 d | 0.24 c | 0.24 c | 39.32 b | 1.34 d | 52.55 c |
|  |  | TS1 | 2.31 c | 2.11 c | 0.33 b | 0.31 c | 0.30 b | 0.27 b | 41.27 a | 1.38 c | 56.92 b |
|  |  | JM26 | 2.9 b | 2.95 b | 0.33 b | 0.34 b | 0.33 a | 0.33 a | 38.18 bc | 1.55 b | 59.25 b |
|  |  | JM22 | 3.41 a | 3.45 a | 0.37 a | 0.37 a | 0.33 a | 0.34 a | 37.97 bc | 1.72 a | 65.22 a |
|  | 150 | BM1 | 2.39 d | 3.22 e | 0.19 d | 0.26 e | 0.18 d | 0.24 d | 22.60 e | 1.32 e | 29.74 e |
|  |  | JN2 | 3.12 c | 3.79 d | 0.24 c | 0.29 d | 0.21 c | 0.25 d | 25.85 d | 1.39 d | 35.96 d |
|  |  | TS1 | 4.10 b | 4.78 c | 0.28 b | 0.33 c | 0.25 b | 0.29 c | 27.65 c | 1.43 c | 39.65 c |
|  |  | JM26 | 4.52 b | 5.76 b | 0.30 ab | 0.38 b | 0.27 b | 0.34 b | 30.76 b | 1.40 b | 43.20 b |
|  |  | JM22 | 5.53 a | 7.46 a | 0.31 a | 0.42 a | 0.30 a | 0.40 a | 32.44 a | 1.53 a | 49.75 a |
|  | 225 | BM1 | 2.71 d | 4.78 d | 0.17 c | 0.30 d | 0.17 c | 0.30 c | 18.79 d | 1.06 d | 19.83 e |
|  |  | JN2 | 4.32 c | 5.91 c | 0.24 b | 0.33 c | 0.23 b | 0.31 c | 20.84 c | 1.17 c | 24.30 d |
|  |  | TS1 | 4.98 bc | 6.18 c | 0.27 ab | 0.33 c | 0.25 ab | 0.30 c | 22.03 b | 1.23 b | 27.05 c |
|  |  | JM26 | 5.66 ab | 7.96 b | 0.26 ab | 0.37 b | 0.25 ab | 0.35 b | 21.99 b | 1.33 a | 29.18 b |
|  |  | JM22 | 6.34 a | 9.68 a | 0.29 a | 0.44 a | 0.27 a | 0.41 a | 25.22 a | 1.33 a | 33.53 a |
| Y |  |  | *** | *** | *** | *** | *** | *** | *** | *** | *** |
| N |  |  | *** | *** | *** | *** | *** | *** | *** | *** | *** |
| C |  |  | *** | *** | *** | *** | *** | *** | *** | *** | *** |
| Y * N |  |  | * | *** | * | *** | ns | * | ** | *** | * |
| Y * C |  |  | ns | ** | ** | *** | ns | * | *** | * | *** |
| N * C |  |  | *** | *** | ns | *** | ns | * | *** | *** | *** |
| Y * N * C |  |  | ns | ** | ns | ns | ns | ns | *** | *** | ns |

Note: NTA, N translocation amount; NTE, N translocation efficiency; CNTA, contribution rate of N translocation amount to grain; NUtE, N utilization efficiency; NUpE, N uptake efficiency; NUE, N use efficiency. Values represented mean, and different lowercase letters indicated significant differences between treatments at *P* < 0.05. *** indicated *P* < 0.001, ** indicated *P* < 0.01, * indicated *P* < 0.05, and ns indicated no significant difference.

**TABLE S6** Models and parameters describing the sensitivity of N translocation amount (NTA) to N supply in five wheat cultivars in 2017-2018 and 2018-2019.

| Year | Parameter | Cultivar | Model | R^2^ | k |
| --- | --- | --- | --- | --- | --- |
| 2017-2018 | NTA_leaf+stem+chaff_ | BM1 | y = 0.0384x + 1.7020 | 0.9690 | 0.0384 |
|  |  | JN2 | y = 0.0452x + 2.3532 | 0.9444 | 0.0452 |
|  |  | TS1 | y = 0.0523x + 2.8528 | 0.9895 | 0.0523 |
|  |  | JM26 | y = 0.0553x + 2.9004 | 0.9956 | 0.0553 |
|  |  | JM22 | y = 0.0693x + 3.3344 | 0.9999 | 0.0693 |
| 2018-2019 | NTA_leaf+stem+chaff_ | BM1 | y = 0.0306x + 0.8823 | 0.9483 | 0.0306 |
|  |  | JN2 | y = 0.0416x + 1.0162 | 0.9777 | 0.0416 |
|  |  | TS1 | y = 0.0432x + 1.9968 | 0.9398 | 0.0432 |
|  |  | JM26 | y = 0.0550x + 1.9648 | 0.9657 | 0.0550 |
|  |  | JM22 | y = 0.0630x + 2.8591 | 0.9392 | 0.0630 |

**FIGURE S1** Correlation between grain weight (GW), duration of fast-increase period (*T*_fast_), biomass at anthesis, and total N accumulation amount (N_TAA_) at anthesis. *** indicated *P* < 0.001, ** indicated *P* < 0.01, * indicated *P* < 0.05, and ns indicated no significant difference.

**FIGURE S2** Response of mean grain filling rate (*GFR*_mean_) and maximum grain filling rate (*GFR*_max_) to N supply in 2017-2018 (A) and 20218-2019 (B).

**FIGURE S3** Effects of N supply on nitrogen nutrition index (NNI) of five wheat cultivars in 2017-2018 (A) and 2018-2019 (B). Values represented mean ± SE, and different lowercase letters indicated significant differences between treatments at *P* < 0.05.
